# A Machine Learning Approach Involving Functional Connectivity Features to Classify Rest-EEG Psychogenic Non-Epileptic Seizures from Healthy Controls

**DOI:** 10.3390/s22010129

**Published:** 2021-12-25

**Authors:** Giuseppe Varone, Wadii Boulila, Michele Lo Giudice, Bilel Benjdira, Nadia Mammone, Cosimo Ieracitano, Kia Dashtipour, Sabrina Neri, Sara Gasparini, Francesco Carlo Morabito, Amir Hussain, Umberto Aguglia

**Affiliations:** 1 Department of Neuroscience and Imaging, University G. d’Annunzio Chieti e Pescara, 66100 Chieti, Italy; 2 Robotics and Internet-of-Things Laboratory, Prince Sultan University, Riyadh 12435, Saudi Arabia; bbenjdira@psu.edu.sa; 3 RIADI Laboratory, University of Manouba, Manouba 2010, Tunisia; 4 Department of Science Medical and Surgery, University of Catanzaro, 88100 Catanzaro, Italy; michele.logiudice@studenti.unicz.it (M.L.G.); sabrina.neri@studenti.unicz.it (S.N.); s.gasparini@unicz.it (S.G.); u.aguglia@unicz.it (U.A.); 5 SE & ICT Lab, LR18ES44, ENICarthage, University of Carthage, Tunis 2035, Tunisia; 6 DICEAM Department, University “Mediterranea” of Reggio Calabria, 89100 Reggio Calabria, Italy; nadia.mammone@unirc.it (N.M.); cosimo.ieracitano@unirc.it (C.I.); morabito@unirc.it (F.C.M.); 7 School of Computing, Edinburgh Napier University, Edinburgh EH11 4BN, UK; K.Dashtipour@napier.ac.uk (K.D.); A.Hussain@napier.ac.uk (A.H.); 8 Regional Epilepsy Center, Great Metropolitan Hospital “Bianchi-Melacrino-Morelli” of Reggio Calabria, 89124 Reggio Calabria, Italy

**Keywords:** psychogenic non-epileptic seizures, power spectral density, phase lag index, rest-machine learning-based diagnosis, EEG-based machine learning techniques for PNES

## Abstract

Until now, clinicians are not able to evaluate the Psychogenic Non-Epileptic Seizures (PNES) from the rest-electroencephalography (EEG) readout. No EEG marker can help differentiate PNES cases from healthy subjects. In this paper, we have investigated the power spectrum density (PSD), in resting-state EEGs, to evaluate the abnormalities in PNES affected brains. Additionally, we have used functional connectivity tools, such as phase lag index (PLI), and graph-derived metrics to better observe the integration of distributed information of regular and synchronized multi-scale communication within and across inter-regional brain areas. We proved the utility of our method after enrolling a cohort study of 20 age- and gender-matched PNES and 19 healthy control (HC) subjects. In this work, three classification models, namely support vector machine (SVM), linear discriminant analysis (LDA), and Multilayer perceptron (MLP), have been employed to model the relationship between the functional connectivity features (rest-HC versus rest-PNES). The best performance for the discrimination of participants was obtained using the MLP classifier, reporting a precision of 85.73%, a recall of 86.57%, an F1-score of 78.98%, and, finally, an accuracy of 91.02%. In conclusion, our results hypothesized two main aspects. The first is an intrinsic organization of functional brain networks that reflects a dysfunctional level of integration across brain regions, which can provide new insights into the pathophysiological mechanisms of PNES. The second is that functional connectivity features and MLP could be a promising method to classify rest-EEG data of PNES form healthy controls subjects.

## 1. Introduction

Although decades of intensive research, one of the most clinical challenges is related to how to treat the subjects having the symptom of paroxysmal episodes of loss of consciousness or altered awareness. Many clinical disorders share this alarming symptom. Among them, the disorder of Psychogenic Non-Epileptic Seizures (PNES) is still lacking research efforts to better understand it. It is characterized by rapid convulsive changes in behavior or consciousness that mimic epilepsy but without the electrophysiological (EEG) bio-markers of the epileptic seizure in EEG readouts. PNES have been associated with a broad range of comorbid neurological and psychiatric disorders, as well as with both physical and sexual abuse in earlier life or with complex disorders in alcoholism, anxiety, or depression [1,2]. PNES could also be related to a positive family history of psychiatric disorders [3]. The genetic risk of PNES shares signs with other psychiatric disorders (including epilepsy, depression, anxiety, and neuroticism) with an overlap in morphological and functional characteristics [4]. The incidence of PNES in the worldwide population has been estimated in Reference [5], and its prevalence, in a huge range of heterogeneous populations, has been estimated in Reference [6]. The investigation of these medical cases, despite extensive and exhaustive clinical inquiries, may remain unfathomable because they largely focused on an insufficient clinical history of the events’ description. Furthermore, the lack of an alternative and robust electrophysiological marker reduces the effectiveness of disentangling the PNES disorder from healthy control subjects in rest EEG readouts.

In well-equipped clinical facilities, the diagnosis of PNES can be strengthened using video-EEG monitoring, with particular mention for those cases where a psychogenic event is captured during spontaneous occurrences or externally triggered episodes [7]. However, video-electroencephalography analysis is expensive, ethically controversial, not commonly available, time and resource-consuming, and, finally, also prone to bias due to subjective interpretation. In spite of this, clinicians routinely face a demand for differential diagnoses of PNES in an EEG readout free from electrophysiological features. An early and accurate diagnosis of PNES represents an important challenge for patients, proxies, and caregivers. In fact, under-diagnosis (or epilepsy misdiagnosis), patients may be subject to a risk of inappropriate and potentially harmful treatment, with a possible impact on patients’ health and placing high burdens on healthcare systems [8]. This makes discrimination of PNES via EEG readout a challenging task for clinicians, especially for cases of false alarms.

This paper proposes a methodological pipeline based on power spectral density (PSD) and phase lag index (PLI). Here, the PSD is used to identify signatures of spectral behaviors, whereas the PLI is used to analyze inter-dependencies between two anatomically-distinct electrophysiological time-series. Furthermore, we used machine learning techniques to test if our graph-driven indices dataset is suitable for an automatic diagnosis of PNES in the rest EEGs. The first goal is to investigate the mechanism underlying information transfer among different brain locations. The repertoire of spontaneous resting-state cortical activity emerges either as a result of the embedded oscillatory activity of neurons and neuronal populations or as an ongoing dynamically changing in the cluster of interconnected neurons [9]. Recent studies on inter-regional brain network communication showed that neural activity is characterized by dynamic fluctuation and synchronization of oscillatory activity between neuronal populations within a millisecond of delay [10,11,12]. Neurophysiologically, the intrinsic good hallmarks of brain cooperation rely on the prowess of multiple functionally specialized areas that interact rapidly and effectively. Thus, if two or more cortical generators promote neural oscillations synchronized in frequency and/or in phase, a higher gain of information is transferred; thus, a more efficient communication occurs. Hence, an emerging idea is that a healthy brain requires an optimal balance between functional integration and functional differentiation in cortical networks. To this end, studying the rhythmic and phase characteristics can reveal the neural phenomena that underlies in PNES disordered, such as a potential transient inter-regional synchronization. To date, a substantial amount of evidence exists for different brain diseases, but, differently from the former, the rest-EEG data of PNES have not been extensively investigated so far. In terms of spontaneous scalp EEGs, a general trend is to investigate the redistribution of power in the frequency bands to find peaks of frequencies or dominant bands. Fourier-based power spectral density, to characterize the frequency distribution of resting-state PNES via EEG data, was used only in References [13,14]. Functional connectivity within particular resting-state networks can be used to study local organization and global integrated and spatially distributed neural areas. In the literature, we found few EEG studies that have focused on derivative tools from graph theory to characterize the synchronization topologies of the neural network of PNES brain systems [10,11,15,16]. Noteworthy is that we found different techniques applied to study the whole-head neural network synchronization of PNES, including coherence analysis [11], multivariate phase synchronization [15], synchronization likelihood [17], and functional connectivity [12,18,19,20], with somewhat contrasting results. According to previous studies, we find two major critical issues in the functional brain network of PNES that need to be further investigated. First, despite different studies, there is no gold standard on the binarization approach to estimate functional network interaction [21]. In functional network analysis, a threshold is often applied to the correlation/phase coupling matrix to remove links with very low strength. Choosing a threshold is important because it affects the impact of the density of the connection in network topology [21] and the network topology interpretation. Second, there are conflicting results in connectivity analyses of PNES. These differences could be related to the impact of subjects and environment and may be due to the use of different methods, such as electrode density, reference technique, coupling methods, binarization approaches, etc. In addition, we aimed to deal with the following points:

- Investigating possible intrinsic alterations in resting-state brain networks oscillations of patients with PNES using PSD analysis.
- Determining whether functional connectivity alterations of PNES subjects could be associated with specific areas which lead to regional network dysfunctions in local oscillations, as well as inter-regional synchronization.
- Investigating a machine learning approach, involving rest EEG-based functional connectivity features, to disentangle PNES from non-PNES subjects.

The remainder of the paper is structured as follows: first, in Section 2, we review the related works in PNES functional analysis. In Section 3, we discuss and provide details on the proposed method, including data preprocessing and the techniques used. Next, in Section 4, we present three classification algorithms for PNES versus HC diagnosis. In Section 5, we present our results in terms of PSD analysis, graph measures, and ML classifiers’ performances. In Section 6, we discuss our results with a comparison with the state of art; finally, Section 7 concludes with take-home messages and future directions.

## 2. Related Works

During the last few decades, a wealth of studies have focused on scalp-EEG data, recorded in resting-state condition, to identify criteria that can differentiate PNES disorders based on its phenomenology [22]. Many of these studies provide a window into neural network oscillations [12,15], functional connectivity [16], loss of integration [23], and dissociation (lost of coherence) [18]. Additionally, in many of these studies is hypothesized that PNES symptoms could be related either to neural network instability [10,15,16,24], due to a mechanism of a time-limited interruption in neural networks connectivity and disconnection between cortical and sub-cortical systems [16,25], or a time-limited increment in functional connectivity between limbic and motor regions [12,24]. Knyazeva et al. [15] used multivariate phase synchronization to reconstruct the synchronization of the whole-head topography between cortical regions in PNES. They founded a reduction of prefrontal synchronization across all frequency bands with hyper synchronization over the left frontotemporal, parietal-temporal networks, along with hypo synchronization in both right and left frontal regions. Using a graph-theoretical approach, Barzegaran et al. [10] studied the function of cortical networks in patients with PNES using Laplacian-transformed time-series and cross-correlation methods, and they found that patients with PNES have close to normal local and global connectivity and small-world structure. Xue et al. [11] applied clustering coefficients (local connectivity) and global efficiency to evaluate the coherence of neural networks for patients with PNES. Clustering coefficients and global efficiency were lower in all four frequency bands, but this difference was statistically significant only in the gamma band. Furthermore, Xue et al. found that PNES had decreased long linkage between the frontal region and posterior brain areas in the gamma band. Barzegaran et al. [16] used multivariate phase synchronization to grapple lagged functional connectivity between cortical and subcortical regions. Their results observed a decreased functional connectivity between the basal ganglia and limbic, prefrontal, temporal, parietal, and occipital regions, in the alpha band. Umesh et al. [17] performed power spectral and lagged phase synchronization analysis in the gamma band using sLORETA software. Umesh’s paper displayed a higher power spectrum in the gamma band in the right temporal region. In addition, the authors found a decreased gamma band in the right parietal cortex, posterior cingulate cortex and superior temporal gyrus. The analysis of connectivity showed a reduced intracortical lagged coherence between the right posterior cingulate gyrus and right middle temporal gyrus. Meppelink et al. [26] found EEG spectral power changes in PNES before seizure events. They also found that PNES subjects showed a decrease in beta power (desynchronization) at 5–6 s before the PNES event. Arikan et al. [27] used a quantitative EEG (qEEG ) and Fast Fourier Transformation (FFT) to investigate the spectral power across all frequency bands in adults with PNES. In this paper, the authors found increased power around the *C3* EEG scalp sensor in the beta band, and, additionally, statistically meaningfully increased gamma power was found in the P3 sensor. Amiri et al. [28] applied a graph network analysis for binary functional connectivity by extracting graph-theoretical measures, such as a nodal degree, in cortical and subcortical regions of the brain. Their experimental findings suggest that the functional connectivity can be altered in individuals with PNES. Areas with low connectivity may be involved in emotion processing and movement regulation, whereas areas with higher connectivity may play a role in the inhibition of unwanted movements and cognitive processes. In this context, it is worth mentioning that no previous studies addressed a machine learning framework involving EEG-based functional connectivity to diagnose rest-PNESs versus rest-HC. We used three well-known machine learning techniques, such as support vector machine, linear discriminant analysis, and Multilayer perceptron, and tested their performances by Precision, Recall, $F_{1}$ score, and Accuracy.

## 3. Materials and Methods

### 3.1. Participants

In this study, we retrospectively reviewed EEG datasets of 80 patients (20 males and 60 females, aged 18–68 years, mean 38.41 years, and Standard deviation (SD) 16.80), who were hospitalized in the Regional Epilepsy Center, Great Metropolitan Hospital Bianchi Melacrino Morelli, Reggio Calabria, Italy, during the years 2016–2019. All data was collected as part of a routine clinical workup. The protocol of the study was approved by the Medical University of Magna Graecia of Catanzaro (Italy), and all the analyses were performed under the approved guidelines. The collected data have been cleared of identification references related to the patients. The subjects with PNES were selected based on the 2013 ILAE Non-Epileptic Seizures Task Force Recommendations [29].

Within the studied group, 60 of the subjects recruited from inpatient or scheduled clinical follow-up were excluded for use of psychotropic drugs (*n* = 40), left-handedness (*n* = 12), or because EEG traces could not be evaluated due to the strong presence of artifacts (*n* = 8). Finally, 20 EEGs from 20 patients with PNES (7 males, mean 35.05 years, SD 13.07) were included. Three inclusion criteria were considered: (1) at least no single typical PNES episode was recorded by video EEG; (2) patients had no clinical history of brain disease; (3) patients had no obvious irregularities in structural Magnetic Resonance Imaging (MRI) examinations. In addition we excluded: (1) subjects with neurological comorbidities (e.g., epilepsy); (2) subjects with simulated or true psychiatric disorders (e.g., mood and anxiety disorders, schizophrenia, and psychosis).

We also enrolled healthy right-handed controls (HC) without a history of neurological disease, seizures, or sleep disorders who did not consume caffeine and were not taking any medicine before the EEG recording. The control group comprised 19 EEGs from 19 healthy right-hand subjects (5 males, mean age: 35.05 years, SD 13.07). The choice to limit the study to right-handed subjects was due to the uncertain hemispheric dominance in the left-handed brain, which might have influenced results. The Edinburgh Handedness Inventory [30] was used to measure participants’ handedness (average EHI score = 52.9, SD = 6.1, range = 65–100). The sample comprised only one ambidextrous participant. We focused on a large cross-sectional community sample of 39 adults, in the age range from 18 to 68 years, to overcome limitations related to age-related changes in the EEG spectral features and functional connectivity in a wide frequency band. A sensitivity power analysis conducted on G*Power [31,32] revealed that our cohort size was large enough to have a statistical power (1−β) of 0.80 to detect significant differences (α = 0.05) between subjects with an effect size of 0.65 (Cohen’s d for Welch test).

### 3.2. EEG Recording

The EEGs were collected in an electromagnetically shielded and light-controlled room, where the participants were seated comfortably with closed eyes in a lab chair. During the data acquisition, a technician continuously monitored the subject to avoid drowsiness and light sleep that could disrupt the background EEG rhythm. The data was recorded according to a standard 20-min clinical protocol including rest condition using a 19 channels EEG system (Micromed, Italy), having a frequency response from DC to 100 Hz (which attenuates by 40 dB per decade). The signals were recorded (sampling rate = 512 Hz; online band-pass filter = 0.1–1000 Hz) using analogical to digital converter (ADC) with 12-bit resolution. The average duration of recording was 20.2 min, within a range of 19.5–22.1 min. The recording transducers, Ag/AgCl ring electrodes, were accommodated according to the international 10–20 system with channel layout: Fp1, Fp2, F3, F4, C3, C4, P3, P4, O1, O2, F7, F8, T3, T4, T5, T6, Fz, Cz, and Pz, on G2 (between electrodes Fz and Cz). The electrode–skin impedance was less than 20 kΩ. EEG signals were down-sampled to 256 Hz, digitally band-pass filtered (0.5–200 Hz, 24 dB/octave), and further notch filtered to reduce power line interference. The EEG data were stored on a local server for further analysis.

#### Preprocessing

**Figure 1:**
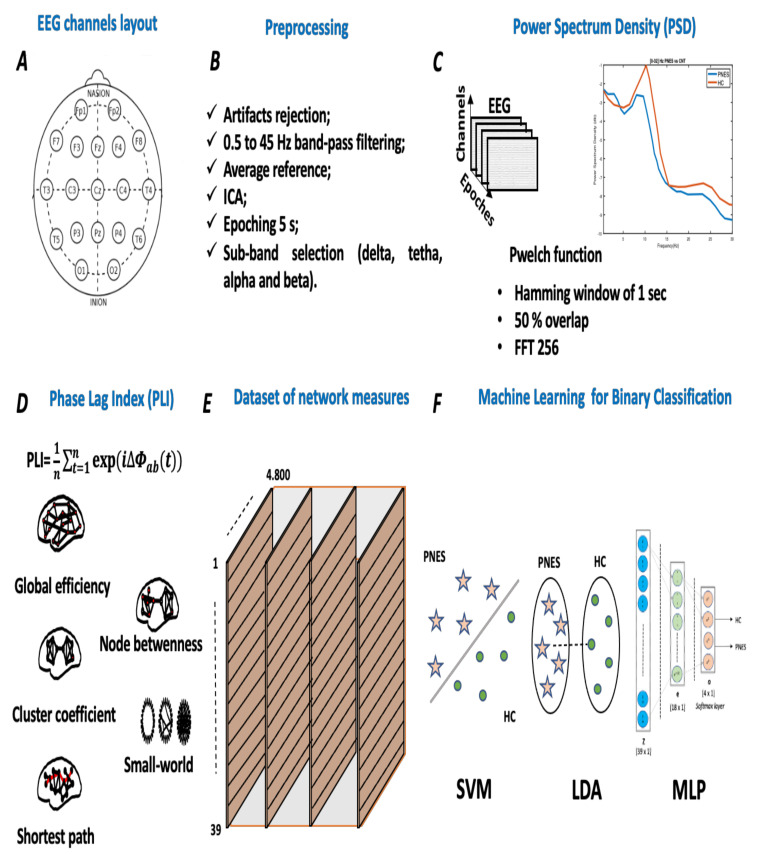
Flowchart of the proposed methodology. Panel (**A**) shows the international standardized 19 scalp EEG channels location. Panel (**B**) depicts the signal preprocessing pipeline used to clean the collected EEG time series. Panel (**C**) highlights the power spectral density processing, where each single EEG channel was fed in the *pwelch* function. Panel (**D**) depicts the EEG-based PLI network analysis. At this stage, each PLI matrix was thresholded and the adjacency matrix was computed. Panel (**E**) lists the indices used to test the performance of the network. In detail, we used global efficiency, node betweenness, cluster coefficient, small-worldness, and shortest path length, such as network indices. Panel (**F**) highlights a schematic representation of three ML classifiers, respectively, support vector machine (SVM), linear discriminant analysis (LDA), and Multilayer perceptron (MLP).

The analysis of participants’ EEG data was performed with custom-built scripts in MATLAB (R2021, Mathworks Inc., Natick, MA, USA) and EEGLAB toolbox [33]. First, the collected EEG time series were visually inspected to detect electrodes with strong artifacts (e.g., jaw clenching or head-scratching); additionally, high-frequency artifacts, such as muscle and high-amplitude slow wave, were rejected using an automatic algorithm with an initial threshold fixed at 80 μV. Secondly, we screened for bad channels (e.g., drifts, and unsettled). If less than 3 electrodes were affected, they were interpolated. Third, the data were further preprocessed by a 0.5 Hz high-pass filter (Butterworth, 3rd order), and then we applied Independent Component Analysis (ICA), using the FastICA algorithm (maximizes the negentropy of the component distributions), to remove independent components (ICs) in concatenated EEG data. ICA is currently used to detect independent components of the brain or artifacts related to eye blinking, eye movement, muscle activity, heartbeat, and instrumental noise. The ICA components are meant to be a linear mixed of EEG recorded data. At this end, let $X_{n}$ × *m* be a matrix of EEG recorded data, where *n* is the number of EEG channels, and *m* is the number of sampling points, as $X\left(t\right)={\left[x_{1}\left(t\right),\;\cdot \;,\;\cdot \;,\;\cdot \;,\;\cdot \;,x_{n}\left(t\right)\right]}^{T}$ and the matrix of d ICs are $S\left(t\right)={\left[s_{1}\left(t\right),\;\cdot \;\;\cdot \;\;\cdot \;\;\cdot \;,s_{m}\left(t\right)\right]}^{T}$, where superimposed *T* indicates matrix transpose. The general ICA model can be written as: X(t)=AS(t)+N(t), where *A* is an [*n* × d] matrix, named mixing matrix, and N(t) is the noise matrix. The *i*th column of *A* contains the weights with which the *i*th IC is distributed across the EEG channels. Here, the $s_{i}\left(t\right)$ corresponds to the *i*th IC time course, and the $A_{i}$ corresponds to the *i*th IC sensor map. To solve the ICA problem, we used the FastICA algorithm (maximizes the negentropy of the component distributions). The ICA algorithm applies to the EEG X(t) signals for artifact-correction can be obtained by a recombination of brain IC as follow: X(t) = Ab ∗ Sb(t). All ICs components were inspected visually and manually selected for rejection (3.41 ± 0.19). Additionally, to validate the selection of ICA components, we applied an ICLabel classification [34]. Fourth, 20 min of artifacts free scalp EEG data were epoched into *M* non-overlapping epochs (of 5 s length). Since sampling rate is ($f_{s}$ = 256), each epoch included *N* = 1280 samples. Here, *M* is equal to 240. Therefore, for every subject under consideration, the recorded epochs $EEG^{n}$ (*n* = 1 … M), sized *n* × N, were stored on a computer and processed one at a time. Fifth, each individual epoch was band-passed using zero-phase Blackman-windowed FIR filters (cut-off frequencies = *f${}_{low}$* (*f* = 0.5 Hz) and *f*${}_{high}$ (*f* = 45 Hz), transition bandwidth = 2 Hz), to select the EEG rhythm range of interest. Finally, for each epoch, we applied a third-order zero-phase shift Butterworth filter to capture the significant electrophysiologic sub-bands: delta (0.5–3.5 Hz), theta (4–7.5 Hz), alpha (8–13 Hz), and beta (13–32 Hz). The proposed pipeline is graphically presented in Figure 1.

### 3.3. Power Spectral Density Analysis

Artifact-free epochs was submitted to one-sided single-trial power spectra periodogram estimation using Welch’s method. The Welch’s method was implemented using MATLAB’s *pwelch()* function, with hamming window, resolution of 0.5 Hz, and overlapping (50%) to obtain power spectral density values (PSD) (μV${}^{2}$/Hz) [35]. The data was converted to power spectral density (PSD) using a decibel transformation (10log10(data)). In our analysis, frequencies from 0.5 to 32 Hz (in 0.5 Hz steps) were returned. The power spectral density was computed using a multi-taper approach, where the time series are sliced into segments, and the average of their periodograms was found. It is a non-parametric approach that represents the auto-correlation function between the signal $x_{i}$ with a length *L* defined as follows:

(1) $$P\left(f_{sb}\right)=\frac{T_{s}}{L}|\sum_{t=0}^{L-1}{\left|x_{i}e^{-2\pi ft}\right|}^{2},$$

where *f* ranges in the interval [$-\frac{1}{2T_{s}}$,$\frac{1}{2T_{s}}$]. Now, the $th_{epoch}$${EEG}^{\epsilon }$ is multiplied by a window function $\omega_{t}$, and the modified periodogram is obtained as follows:

(2) $$\hat{P}\left(f_{sb}\right)=\frac{T_{s}}{L}|\sum_{t=0}^{L-1}{\left|\omega_{t}x_{i}e^{-2\pi ft}\right|}^{2}.$$

Here, the PSD of the $th_{epoch}$ ${EEG}^{\epsilon }$ was computed by using the modified periodogram with a hamming function, as it achieves good resolution and is well-suited for biomedical signals analysis. We use an EEG segment equal to 5 s length. This type of windowing is generally used to reduce spectral leakage and to smooth the power spectrum [36]. At the end, given the *i*th (*i* = 1, ..., 19) EEG dataset, with *M* = 240 EEG epochs of 5 s, we performed a PSD analysis. The final dataset was organized in channel × frequency × trials matrix.

### 3.4. Graph Analysis

Graph theory offers a method to study the relation between network structure and function, regarding, for example, measures of efficiency, robustness, cost, and growth. A graph is a representation of a network, which is indicated by its nodes (‘vertices’) and connections (‘edges’). Graphs can be characterized by several parameters and principally by a clustering coefficient (*CC*), shortest path length (*SPL*), node betweenness (*NB*), global efficiency (*Ge*), and small-world (*SW*). The small-world network architecture could be of primary importance for cortical dynamics because it represents a balance between local information processing and rapid sharing of this information with other regions. The common parameters to describe the graphs are the clustering coefficient (for segregation) and the path length (for the integration). The former is a measure of the local connectivity of the graph, whereas the latter is an index of overall connectivity. The small-world networks organization, instead, is focusing on an optimal balance between local specialization (segregation) and global integration. Therefore, we describe measures of functional integration and segregation and quantify the importance of individual brain regions in networks to PNES. Graph analysis was implemented using the Brain Connectivity Toolbox [37].

### 3.5. Phase-Locking Index Analysis

In this paper, we apply PLI [38] to quantify the asymmetry in the distribution of the phase difference across all pairwise combinations of EEG sensors. PLI analysis result in a 19 × 19 matrix (19 = number of EEG channels). PLI is a reliable measure of phase synchronization that results invariant against typical issues of common sources, volume conduction, and/or active reference electrodes [39]. The idea around PLI is to discard the index of phase differences in the range of 0 to π when the distribution index is centered around a phase difference of zero. Asymmetry of the phase difference distribution means that the likelihood interval −π < ϕ < 0 is different from the likelihood interval 0 < ϕ < π. The difference in likelihood interval means that there exists a phase difference or time lag. The asymmetry index of phase difference distribution between time series can be obtained in Δϕtk, where *k* = 1 … N as:

(3) $$PLI=|\langle sign\left[sin\left(\Delta \phi \left(t_{k}\right)\right)\right]\rangle |,$$

where $\Delta \phi (t_{k})$ represents the phase difference, and the sign function denotes the average over time. Here, a PLI of zero stand for no phase coupling between two-time points centered in the range 0 mod π, whereas a PLI value of one stand for a phase difference $\Delta_{\phi }\left(t_{k}\right)$ in a range different from the former. Different from other phase synchronization measures, such as phase coherence and the imaginary component of coherency, PLI is much less affected by the influence of common sources (volume conduction) and active reference electrodes [39]. In this paper, PLI analysis was performed on each epoch${}_{i}$ by measuring the functional connectivity (${PLI}_{\left(i,j\right)}$ ) between all possible pairs of electrodes *i* and *j*. Considering n=19 sensors, there are n(n−1)/2=171 possible pairs of channels. The ${PLI}_{ith}$ matrix, was determined for every epoch (with e = 1, ⋯, ⋯, ⋯, 240).

The related (symmetric) connectivity matrix was generated by associating each off-diagonal (*i*,*j*), with the corresponding value of ${PLI}_{\left(i,j\right)}$. For each subject, the original artifact-free EEG was subdivided into non-overlapping segments, here named (epochs). For each epoch, five 19 × 19 connectivity matrices have been derived, respectively, in delta, theta, alpha, and beta, as well for the wide-band. To obtain a solid measure of functional connectivity, we used a 5 s length window to be within at least 4 oscillatory cycles. The lower edge was set to 0.5 Hz. Next, the network measures used in this paper were calculated based on the 19 × 19 adjacency matrix. For each adjacency matrix, we calculated the graph *G* for each thresholded PLI matrix. Each graph is represented as G=(N,W), where *N* is equal to the number of EEG sensors, and *W* is equal to $w_{ij}$; thus, *W* is the N×N symmetric matrix $w_{ii}=0$ and $w_{ij}$. Finally, the PLI index is determined between electrode *i* and *j*.

#### 3.5.1. Network Parameters

To perform network analysis based on PLI measure, the first step was to extract binary graphs, normalized to the interval 0–1, from the weighted connectivity matrices. However, it is noteworthy to mention that not all the weighted links in the initial connectivity matrices are significant; thus, it is better to remove the non-significant branches and minimize the noise level. There are no exclusive methods for binarizing the connectivity matrices. A simple approach to binarizing a weighted connectivity matrix is to apply a threshold *th*. Using this approach, when a link ${PLI}_{i,j}$ has a weight higher than *th*, the corresponding entry value of the adjacency matrix is one, or zero otherwise. The common pitfalls with the binarization approach are that several valid threshold values can be found to extract important branches. To overcome this challenge, the threshold value was often kept in a fixed range. However, there may be individual variations in the functional connectivity since some subjects might have higher average connectivity than others. In those cases, the same threshold values, applied to all subjects, may lead to different network densities. Many of the network topological properties can be biased by the binarized approach. Thus, to avoid these confounding factors, one can study the network properties as a function of density instead of the threshold. We analyze the impact of different threshold values in network binarization methods. We applied a binarization method in the range 0.05 to 1 with a step size of 0.5 to test network consistency and robustness. We shuffled all the adjacency matrices, computed in each sub-band at each *th* value, before we applied a permutation test. We repeated this procedure using a simulation with 1000 iterations and extracted the clusters from each permutation to compare the latter with the original dataset. Our statistical analyses find that *th* = 0.05 is a value of knee, a good trade-off for network consistency. To obtain normalized network measures, the entry PLI values were normalized between 0 and 1, then divided by the average obtained from a set of 101 random graphs (obtained by randomization of all actual matrices) with the same number of nodes and connections as the actual graphs. We performed a PLI analysis between each pair of electrodes, for each subject, within each sub-bands. Thus, given a sub-band *sb* (delta, theta, alpha, and beta) and the corresponding EEG signals ${EEG}_{sb}$ (as reported in Section 3.5), in an generic epoch *M* under analysis, the ${{{PLI}_{\left(i,j\right)}}_{sb}}^{1,\ldots M}$ is calculated between every pair of electrodes *i* and *j*. Each ${{{PLI}_{\left(i,j\right)}}_{sb}}^{M}$ is thresholded, and the adjacency matrix is computed.

### 3.6. Graph Metrics

In the literature, we found a different graph theory metrics, but not all of them are robust enough for studying the brain network functions. Here, we take into consideration several neurobiologically relevant network measures related to brain functions including information, segregation, and integration. Indeed, since EEG signals are interdependent, spurious links may be generated by applying PLI measures to estimate connectivity from them. We tested the connectivity matrices with a threshold of 0.5 that provides an optimal trade-off between reducing spurious connections and retaining true connections. The graph analysis is explored in greater depth in Section 5.2. Functional integration in the brain represents the ability to integrate information among distributed brain regions. Measures of integration or differentiation are developed to characterize the communication among brain regions. Measures of communication between cortical areas are commonly based on the concept of a path. The path is a sequence of distinct nodes and links, and represents potential routes of information between pairs of brain regions. We used several different graph measures including average shortest path length, global efficiency, cluster coefficients, small-worldness, and node betweenness.

#### 3.6.1. Averaged Shortest Path Length

The path length is a measure of functional integration of the network and represents the potential for functional integration between brain regions. The shortest path length between two nodes is the minimum number of edges between two nodes [40]. The average shortest path connecting any couple of nodes in the graph is the shortest path represented as follows:

(4) $$\lambda =\frac{1}{n(n-1)}\sum_{i\neq j}d_{i,j},$$

where $d_{i,j}$ stands for the shortest distance between nodes *i* and *j*. The averaged shortest path length is a graph measure, which describes how well its elements are integrated/interconnected. A common drawback of the characteristic path length is that, if any pair of nodes *i* and *j* are not connected through any path, the corresponding shortest path length value is $d_{i,j}$ = *∞*. Here, we refer to the averaged shortest path length as (*SPL*).

#### 3.6.2. Global Efficiency

Differently from the characteristic path length, the global efficiency (Ge) is a measure of network performance based on its global topology. The global efficiency often increases by increasing the network density (e.g., the number of connections), which is inversely related to the topological distance between nodes, and it measures the global information across a network. Global efficiency is a graph index defined as the inverse of the average path length among all nodes [41].

Formally, it is calculated as follows:

(5) $$Ge=\frac{1}{n(n-1)}\sum_{i\neq j}\frac{i}{d_{ij}},$$

where *n* is the network size, and $d_{ij}$ is the average distance between the node i and j in the network. The global efficiency is a measure of the integrated information between distributed nets and stands for the overall network capacity to transfer information in parallel.

#### 3.6.3. Clustering Coefficient

The clustering coefficient is a measure of local structure in a network. Let *i* be a node in the network that has $d_{i}$ edges connected to other nodes. It is a measure of functional segregation, that represents the ability of processing that occurs within inter-connected brain regions. If every neighbor is connected to every other neighbor in the network, the number of edges between them would be $d_{i}\frac{2e_{i}}{d_{i}\left(d_{i}-1\right)}$ [42]. The ratio of the number of edges and the maximum possible number of edges between those neighbors is computed as:

(6) $$c_{i}=\frac{2e_{i}}{d_{i}\left(d_{i}-1\right)},$$

where $c_{i}$ ranges between [0,1] and represents the extent of local cliquishness. In a network, a clustering coefficient, e.g., 1 or close to 1, indicates that its neighbors are well-connected and reachable from many paths; thus, the network is robust to random failures. The clustering coefficient of the entire network is an aggregate measure that is the sum of the clustering coefficients at each node. It can be computed as:

(7) $$CC=\frac{1}{n}\sum_{1\in N}C_{i}=\frac{1}{n}\sum_{1\in N}\frac{2t_{i}}{k_{i}\left(k_{i}-1\right)},$$

where $CC_{i}$ represents the cluster coefficient of the node *i*, $k_{i}$ is the degree of node *i*, and $t_{i}$ denotes the number of edges between pairs of nodes, *j* and $j{}^{\prime }$, that are both connected to *i*. Here, *n* is the number of nodes in a network. If $C_{i}$ = 1, every node in the network is connected to every other node. The number of the connections around a node *i* can be calculated as $k_{i}$, ($k_{i}-1$)/2, where $k_{i}$ is the degree of node *i* [43].

#### 3.6.4. Small-Worldness

The human brain consists of complex and specialized areas for sharing and integrating information [44]. The connections between cortical areas are not random but organized in a so-called small-worldness network topology. Integration and segregation properties combine functional specialization with higher-order processing, such as multi-sensory integration, cognition, and executive functions that require large-scale integration. The small-worldness reflects an optimal balance of functional integration and segregation [40]. The small-worldness network measure is defined as:

(8) $$SW=\frac{C}{C_{rand}}\frac{E}{\lambda_{rand}},$$

where C${}_{rand}$ and $\lambda_{rand}$ are the clustering and path length coefficients of a random network with an equal number of nodes. The functional integration of brain regions (hubs) plays a crucial role in network interactions and communication across brain areas.

#### 3.6.5. Node Betweenness

Node betweenness (NB) is a measure of the centrality of a node in a network and is calculated as the fraction of shortest paths between pairs of nodes that pass through the node of interest. Node betweenness is used to measure the influence of a node on the propagation of information through the network [45]. NB is defined as:

(9) $${\mathrm{NB}}_{i}=\sum_{i\neq j}\frac{\lambda_{ij}\left(i\right)}{\lambda_{ij}},$$

where $\lambda_{i,j}$ is the number of shortest paths between nodes **i** and **j**, and $\lambda_{i,j}\left(i\right)$ is the number of shortest paths making use of the node **i**.

### 3.7. Statistical Analysis

To calculate the statistically significant differences between PNES and HC, we used a non-parametric cluster-based permutation test to address the multiple comparison problem. We investigated the relationship between brain areas and neural rhythms. First, we applied a Shapiro–Wilk test to analyze the distribution of our data. The null hypothesis stands for independently distributed data at different thresholds. Thus, we will accept the null hypothesis if the *p*-value is higher than the chosen significant α level. Additionally, we performed a two-tailed permutation test, using a family-wise alpha1 level of 0.05 and alpha2 of 0.001 to construct clusters of significant spatial *t*-value maps. Separately, we represent our results in a box plot to highlight the differences between frequencies and brain areas between PNES and HC. For the permutation test, we shuffled all epochs and split them into two datasets. Next, we calculated cluster-level statistics and then the probability to estimate cluster-level *p*-values for each area and frequency. If the PSD data were the input of the cluster-level statistics, the output was a *p*-value for each cluster-level statistic. The cluster-level *p*-values were corrected and approximated using a permutation test. We constructed a box plot of the cluster-level statistics with the calculated probability of cluster-level *p*-values for each EEG rhythm under analysis. The analysis was run with MATLAB 2021b and its associated toolboxes.

## 4. Classification

The first goal of our analysis was to assess the ability of functional connectivity analysis to find important differences between HC and PNES. Further, we tried to use common machine learning algorithms to classify functional connectivity network indices as belonging to PNES or HC class. A database of 20 PNES and 19 HC was submitted to graph analysis, and five indices were computed for each epoch (M = 240) and each sub-band (*sb* = 4) at the threshold *th* = 0.05. In the end, the final size of our database for each subject is epoch × network indices × sub-band. Before applying a machine learning classification, we performed a dataset preparation. It is well known that machine learning algorithms have better performances when the dataset is cleaned of outliers and normalized [46,47]. For this reason, we have applied a data preparation stage. We performed an outliers detection using Median Absolute Deviation Estimator [48] for a dataset with a uniform scale.

### 4.1. Dataset Preparation

We performed outliers analysis, and then we split our dataset *D* in $D_{test}$ and $D_{training}$. In this paper, we used 8-fold cross-validation on ($D_{training}$ = 70%), whereas the remaining 30% was used as a test. Our 2D dataset has shape of 39 rows and 4.800 columns, for a total size of 187.200 instances, designed with an additional column for class labeling. Here, HC is labeled as 0, and PNES as 1.

### 4.2. Proposed Machine Learning Classifier

In order to test the hypothesis of a robust, fast, and early diagnosis of PNES, derived by our functional connectivity indices, we apply three well-known Machine Learning Classifiers (support vector machine, linear discriminant analysis, and multilayer perceptron (MLP)).

**Figure 2:**
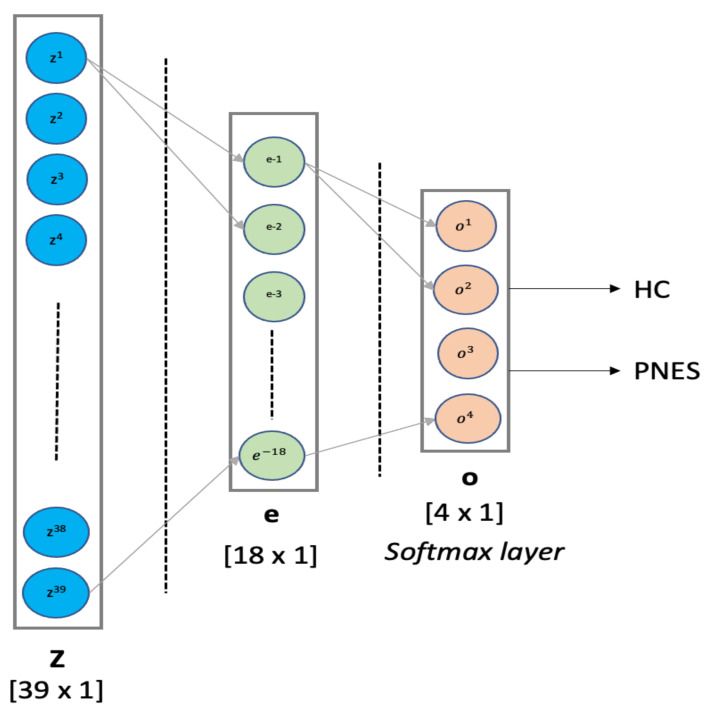
MLP architecture. Our MLP architecture comprises one hidden layer with 18 units, followed by a *softmax* layer, for binary classification.

- *SVM classifier*: SVM technique is a computer algorithm that learns, based on a statistical theories, labels assigned to objects [49,50]. SVM technique attempts to find a hyperplane that provides the best separation between classes of points. In this study, a SVM classifier with a linear kernel is implemented. The mathematical background on SVM is reported in detail in Reference [51]. SVM classifier is suitable to work with 2D datasets; thus, it was fed with an R (raw) ∗ C (column) matrix set. Here, (R = 39) and (C = 4.801). However, after training/test dataset selection, here, 70% for training and 30% for testing, it was submitted for 8 k-fold cross-validations. The SVM classifier was implemented in python using scikit-learn packages. The parameter C is a hypermeter in SVM used to control the error of class separation. Here, we used C = 0.01.
- *LDA classifier*: Linear Discriminant Analysis uses a statistical methods applied for data classification and dimensionality reduction. LDA reduces the data dimensionality in order to improve the class separability. LDA projects clusters of data into lower dimensional space to increase class separability by decreasing intraclass differences. More mathematical detail on LDA background is reported in Reference [52]. Our LDA classifier is suitable to work with 2D datasets; thus, it was fed with an R (raw) ∗ C (column) matrix set. Here, (R = 39) and (C = 4.801). The LDA classifier was implemented in python using scikit-learn packages.
- *MLP classifier*: Multilayer Perceptron is a supervised feed-forward neural network commonly used for classification and regression tasks [53,54]. We designed an MLP classifier with two hidden layers with 18 and 4 neurons, respectively. The first hidden layer was designed with *ReLU* function, whereas the latter hidden layer implements a *softmax* for binary classification. The training procedure was submitted to k-fold cross validation. MLP was implemented in python using scikit-learn packages. Our MLP architecture is depicted in detail in Figure 2. In this paper, the MLP is trained using supervised learning mode for $10^{3}$ epochs on a MacBook Pro 2.2 GHz Intel Core i7 quad-core (training time ≈ 480 s). The features vector (sized 1 × 39) is used as input to a MLP with 2 hidden layers of 39 and 18 hidden units, respectively. The *ReLU* is used as activation function for each hidden neuron. The network ends with a *softmax* output layer to perform a binary classification task: PNES versus HC. The architecture here was referred as ${\mathrm{MLP}}_{\left(39,18\right)}$.

## 5. Results

### 5.1. Relative PSD Analysis

**Figure 3:**
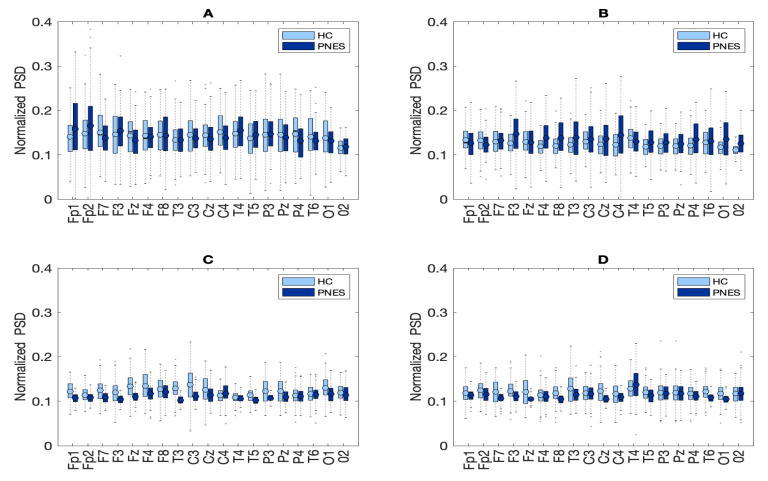
Box plots of normalized PSD values between pairwise of similar sensors in PNES versus HC. Panel (**A**) depict the PSD results of pairwise comparison in delta band, panel (**B**) in theta, (**C**) in alpha, and (**D**) in beta band.

**Figure 4:**
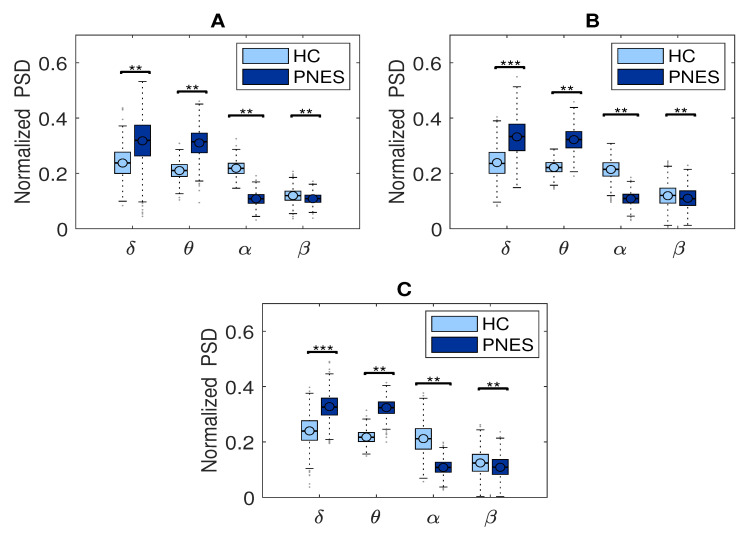
Relative normalized PSD, averaged over the 19 electrodes, in four frequency bands for frontal (see panel (**A**)), central (see panel (**B**)), parieto-occipital area (see panel (**C**)) between PNES and the control group. The horizontal mark within each box represents the median, the edges of the box represent the first and third quartile. The horizontal line denotes significant difference between groups, where ** stand for *p* < 0.05, and *** for *p* < 0.001. The PSD measures was computed in PNES and control in the delta, theta, alpha, and beta frequency bands.

In the present resting-state EEG study, for each subject and each epoch, we have extracted a set of features. In particular, the relative *delta*, *theta*, *alpha*, and *beta* power bands were computed for each channel as the ratio between the sum of the original PSD (computed using the Welch method in the whole band 0.5–32 Hz (total power)). Next, the PSD was split into the preferred frequencies: delta (0.5–4 Hz), theta (4–7 Hz), alpha (8–12 Hz), and beta (13–32 Hz). Our power spectral density EEG analysis was also conducted within subjects. Notably, in the delta frequency band, the relative normalized PSD values for PNES ranged between [0.1, 0.3 μV^2^/Hz] *log* normalize values. Furthermore, in beta band, we founded PSD values between [0.025, 0.25 μV^2^/Hz], indicating that most energies focused at lower frequencies when the subjects were resting. Comparing the relative PSD values between the PNES group and the HC (see Figure 3A), we found higher PSD in the delta and theta band for PNES more than HC. Conversely, in the alpha band (see Figure 3B), the PSD results are higher for HC. To infer more insight into the power spectrum results, we performed a post–hoc analysis in four frequency bands and three brain parcellations (Frontal, Central, and Parieto-occipital areas) (see Figure 4). In this paper, we selected (Fp1-Fp2-F3-F4) sensors for frontal area, (C3-C4-Cz-Pz) sensors for the central area, and the sensors (P3-P4-T3-T4-T5-T6-O1-O2-P3-P4) for parieto-occipital. The PNES group was found to have significantly higher PSD in the delta band in the frontal and central area (*p* < 0.05), and in the delta and theta band. Additionally, we found an increased PSD in alpha and beta for HC, more than PNES in the frontal, central, and parieto-occipital areas (*p* < 0.01). In the theta band, post-hoc analysis showed that the PNES group differed as compared to HC (*p* = 0.0082, *p* = 0.0476, and *p* = 0.0076, respectively, for the frontal, central, and parieto-occipital area). Differences were also present in the beta band (*p* = 0.0255 and *p* = 0.0308 and *p* = 0.0043, respectively). In the beta band, we found higher values of PSD for HC compared to PNES. In the delta band, HC showed a lower PSD values as compared to HC (*p* = 0.0262, *p* = 0.342, *p* = 0.0153). In the alpha band, the values of PSD for HC were higher than PNES with (*p* = 0.0032, *p* = 0.00443, and *p* = 0.023, respectively) in all the brain parcellations. Compared to HC, the relative PSD values were increased for PNES in the delta band in the frontal and central areas. In contrast, PSD for alpha and beta bands were increased in the HC more than the PNES subjects in all the parcellations. Compared to HC, the relative PSD values were increased for PNES in the delta band in the frontal and central areas. As illustrated in Figure 4, frontal, the central, and parieto-occipital area displayed a significant PSD difference in the delta and theta bands.

### 5.2. PLI Analysis

Given an ${EEG}^{\epsilon }$ recording (i.e., ϵ = 1,…, 19) and an *M* epochs (*M* = 240 of 5 s each), a ${PLI}_{e}$ matrix is determined according to Section 3.5. Let *G*(*V*, *E*) be a completely weighted graph with a set of *V* nodes ($v_{1}$,$v_{2}$,…,…,$v_{n}$), a set of *E* edges, where each edge ω(u,v) in *E* is an unordered pair of nodes in ω(u,v), and *u* and *v* are the weighting edge connections. The connected thresholded sub graph *G*’(*V*’, *E*’) includes all the edges with weights between a threshold *th*. Here, for each $G^{\prime }{\left(V^{\prime },E^{\prime }\right)}^{\epsilon }$, in each sub-band, we performed network parameters (e.g., λ, *Ge*, *CC*, *SW*, *NB*) as described in Section 3.5.1. Then, for each epoch *M*, we applied Brain Connectivity toolbox routines to extract network parameters. Considering the λ network measure, computed at epoch *M*, four-vectors, one for each band of frequency under analysis, are designed, respectively, for Lambda-HC and Lambda-PNES. Further, to highlight the differences in graph measures, we performed a thresholding analysis at different values of *th*. A different threshold means a graph with a different edge density that leads to infer the degree of network disconnectedness. The disconnectedness of the graphs affects the quantitative values of many network metrics; thus, choosing a good threshold might be a significant challenge in making a fair comparison between networks estimated in healthy volunteers and in PNES. All the graph metrics were initially estimated in the network from 0.05 to 1 with step 0.1% of density. We explored different thresholding values that force graphs to be connected even at sparse densities to address the issue of disconnectedness that can arise as a result of global thresholding. Based on the thresholding approach used when the entry of the PLI matrix (Figure 1) is larger than the threshold, the entry is set to 1, and otherwise 0. It is well known that many network measures are heavily dependent on network density. The approach is, therefore, to binarize the connectivity matrices in a way that each network has the same density [55]. We binarized the matrices using the threshold value of 0.05. The matrix ${PLI}_{ij}$ of 19*19 values was transformed into a binary matrix $A_{ij}$ (Adjacency matrix). Suppose the ${PLI}_{ij}$ value was higher than the threshold of 0.05, the corresponding $a_{ij}$ of the binary matrix $A_{ij}$ is fixed to 1 (link), indicating the group difference of PLI between electrode *i* and *j* was significant; otherwise, it equals to 0 (no Link). Thus, the functional connectivity can be extracted from the PLI matrix, and the functional properties could be further quantified. Furthermore, for each adjacency matrix, we computed network parameters (see Section 3.6). We found that all the network values were statistically significant (*p* < 0.05) in a random permutation test across all subjects within each frequency sub-band at 0.05 of the threshold between HC and PNES. *th* (0.05) (Global efficiency; delta (*p* = 0.045); theta (*p* = 0.00032); alpha (*p* = 0.032) and beta (*p* = 0.0044)); (Shortest path length; delta (*p* = 0.0133); theta (*p* = 0.0024); alpha (*p* = 0.013) and beta (*p* = 0.033)); (Cluster coefficient; delta (*p* = 0.023); theta (*p* = 0.014); alpha (*p* = 0.00023) and beta (*p* = 0.0122)); (Small world; delta (*p* = 0.0022); theta (*p* = 0.032); alpha (*p* = 0.0013) and beta (*p* = 0.042)); (Node betweenness; delta (*p* = 0.014); theta (*p* = 0.023); alpha (*p* = 0.0033) and beta (*p* = 0.023)).

**Figure 5:**
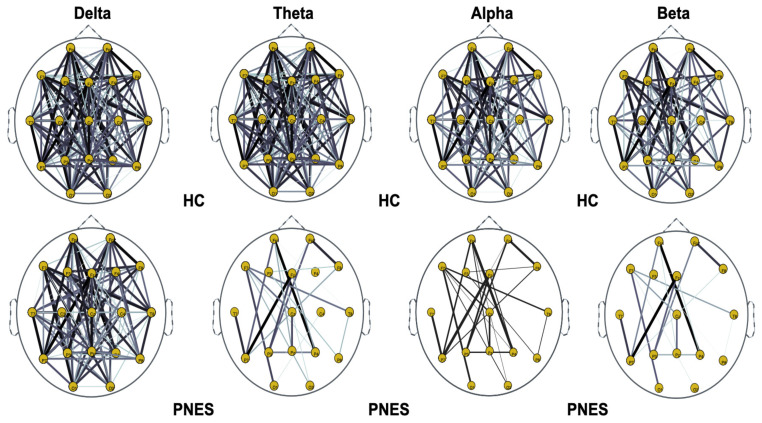
Distribution of PLI for *th* = 0.05 level of the connection strength, in healthy subjects and PNES in delta, theta, alpha, and beta frequency bands. The line thickness highlights path with high value of PLI.

**Figure 6:**
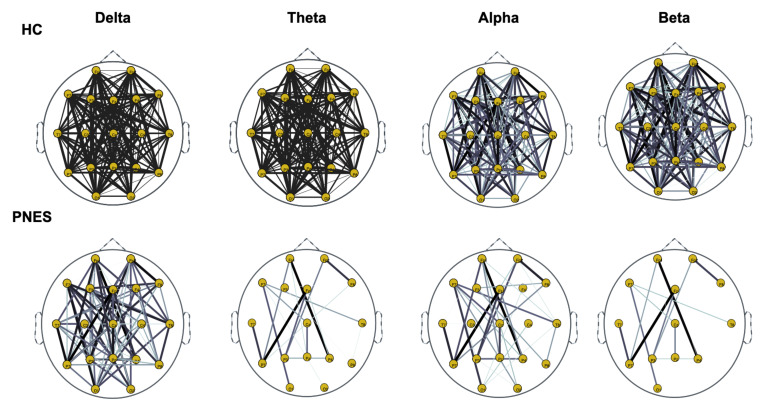
Distribution of PLI for *th* = 0.15 level of the connection strength, in healthy subjects and PNES in delta, theta, alpha, and beta frequency bands.

**Figure 7:**
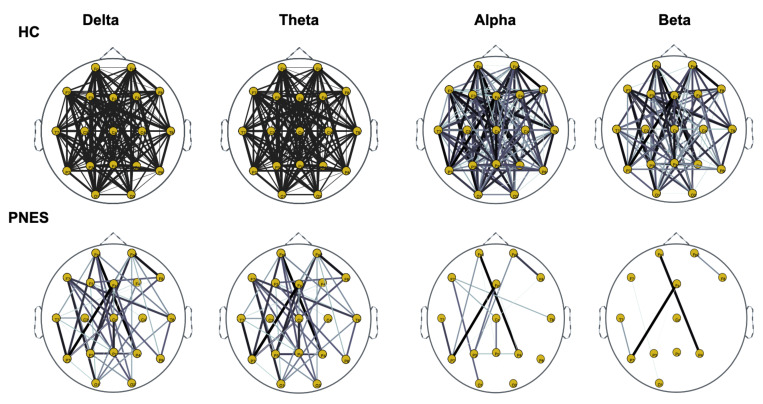
Distribution of PLI for *th* = 0.25 level of the connection strength, in healthy subjects and PNES in delta, theta, alpha, and beta frequency bands.

Next, we compute the symmetry in the distribution of the phase, across all pairwise combinations of scalp sensors, using the phase lag index, considered as a measure of the synchronism of different EEG time series in the frequency domain. In this paper, PLI analysis was applied to all pairwise EEG channels for the PNES and the healthy control group. Figure 5, Figure 6 and Figure 7 show the topographic maps of the relative PLI for PNES and the HC group.

As highlighted in Figure 5, Figure 6 and Figure 7, there are more fully connected areas in HC than the PNES group. PNES group showed high values of synchronization in the delta and theta band, while scattered distributed connections in the frontal, temporoparietal, and occipital areas in the alpha and beta bands. The HC group had a higher synchronization within frontal regions than PNES in delta and theta. We observed higher synchronization within the areas of the frontal and front-to-back regions for the HC group. In the beta band, less synchronization between the frontal, temporal, and parietal regions in the right hemisphere was observed for the PNES group. Lastly, in the alpha band, the synchronization in the left hemisphere was greater than the right hemisphere in the PNES group.

**Figure 8:**
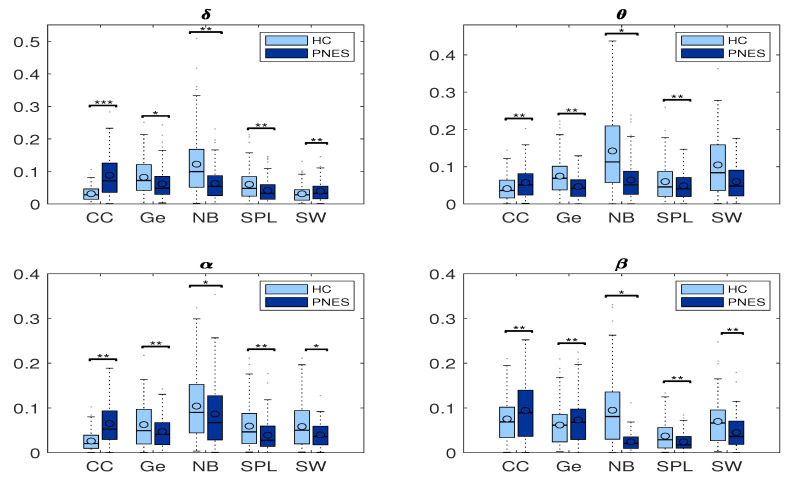
Box plots of network coefficients measured in PNES and HC. Here, we report normalized network coefficients values of: *CC*, *Ge*, *NB*, *SPL*, and *SW* in *delta*, *theta*, *alpha*, and *beta* bands. The horizontal mark within each box represents the median, the edges of the box represent the first and third quartile, and the whiskers extend to the most extreme data points that are not considered outliers, where the symbols ** stand for *p* < 0.05, and *** for *p* < 0.001. The PSD measures was computed in PNES and control in the delta, theta, alpha, and beta frequency bands.

In Figure 8, we highlight the network coefficients measured in PNES and HC.

We further performed a statistical analysis based on two-tailed independent *t*-tests. We highlight group differences in the shortest path length, small worldness, global efficiency, node betweenness, and clustering coefficient at any threshold value among PNES and HC group. Our goal was to find important differences in network measures to infer significant network features. Here, a significance level was taken as *p* < 0.05. Additionally, post-hoc analysis showed that there was a significant influence of threshold at different frequencies on node betweenness, cluster coefficient, and small worldness (all *p*-values corrected for double comparison using Bonferroni correction: *p* < 0.05).

As reported in Table 1, our statistical analysis depicted an overall difference in network indices in central and parieto-occipital areas. Beyond the threshold used to binarize the network, the areas of the brain in which the PNES exhibit a lower values of network parameters were related to the central area and the posterior area. We found a higher statistical variation in the central area more than in the parieto-occipital area. We found global efficiency differences in alpha (*p* = 0.014) and beta (*p* = 0.0011) with a central to posterior trend. Additionally, our results underpinned a Cluster coefficient statistical difference (*p* = 0.0022) in alpha and (*p* = 0.021) in beta for central and parieto-occipital areas. We found more small world differences in the central area than the occipital. We found higher difference between brain areas predominantly in shortest path length; thus, it could be the most discriminative.

#### 5.2.1. Measures of Integration and Segregation

Measures of integration estimate the score of communication between distributed nodes. While the shortest path length (*SPL*) indicates that each node can reach other nodes with a path composed of only a few edges. The global efficiency is an appropriate measure in the case of disconnected networks since the paths between disconnected nodes have infinite length and, thus, zero efficiencies. The global efficiency is the average inverse shortest path length, defined as one of the most elementary measures of the network’s integration, so that this measure may be treated as an indicator of segregation that can share information between distributed regions. The measure of segregation defines the ability of specialized processing within a densely interconnected group of nodes in a specific network (clusters or modules). We also used a measure of segregation, the clustering coefficient, defined as the fraction of the node’s neighbors that are also neighbors of each other. Segregation and integration are interdependent measures. Segregation decreases and integration increases as global pairing increases. When the global coupling is weak, there is high segregation and low integration, and the perturbed nodes are disconnected and it operates in an independent way. Conversely, when the global coupling is strong, the integration is high and the segregation is low because the perturbed nodes are coupled. We found significant differences in clustering coefficient (CC) in the delta, theta, and alpha bands. We found that CC was increased in the delta, theta, and alpha bands for the PNES group (Figure 8). In addition, no significant differences are observed for *Ge* in all four-frequency bands (except *Ge* in the alpha). We found that *Ge* was relatively higher for the HC group than the PNES group in the whole band. As a measure of integration, the shortest path length shades light over the efficiency of global information communication. The shortest path length is a unique sequence of edges that connects two nodes, and its length is given by the number of steps or the sum of the edge with the shortest path length. We found a decrease of the shortest path length in delta, theta, alpha, and beta bands for the PNES group compared to the HC group. In the beta band, the *SPL* value is slightly lower in the PNES group than the HC group (Figure 8). The human brain can be described as a small-world network structured around a large number of spatially distributed network communities with clustered connectivity, in which the local computations are highly segregated. In small-world architecture, integrated and segregated information are promoted by network hubs that arrange efficient communication and information processing.

#### 5.2.2. Measures of Centrality

The centrality is a measure of the relative importance of a node connecting a network in the brain; hence, it is important for the functional integration of the brain. Nodes with high centrality are called hubs. Our results highlighted a decrease of Node betweenness in PNES in delta, theta, alpha, and beta bands more than HC group (Figure 8). Our findings highlighted a loss of network performance.

### 5.3. Binary Classification

The performance of the proposed classifiers, Support Vector Machine, Linear Discriminant Analysis, and Multilayer Perceptrons is measured using traditional metrics: *precision*, *recall*, *F1 score* (or F-measure), and *accuracy*, defined below:

(10) $$Precision=\frac{TP}{TP+TF},$$

(11) $$Recall=\frac{TP}{TP+FN},$$

(12) $$F_{1}score=2*\frac{Precision*Recall}{Precision+Recall},$$

(13) $$Accuracy=\frac{TP+TN}{TP+FN+TN+FN},$$

where *TP* stands for (True Positive) and is the number of labels classified as true while they were actually true; *FP* (False Positive) is the number of labels classified as true while they were actually false.; *FN* (False Negative) is the number of labels classified as false while they were actually true; and *TN* (True Negative) is the number of labels classified as false while they were actually false. In order to estimate the ability of each classifier to correctly classify network parameters, as belonging to HC or PNES subjects, we perform a comparative performance of our proposed machine learning classifier (MLP, SVM, and LDA). In this study, we used a k-fold cross validation that split recursively the dataset into two sets: train set composed of all instances excluded the *i*th, and the test set composed of the *i*th k-fold observation. Our is dataset sized as 39 × 4800, where 39 are the total number of subjects, and 4800 are the instances of our classification dataset. Machine learning algorithms work on a 2D dataset; thus, the second dimension of our dataset turns out to be a flattened version of features extracted in $M_{240}$ epochs × $S_{sb=5}$ × features${}_{f=5}$. The k-fold cross-validation involves using one subject as the test set, and the remaining subjects for the classifier. In our case, we have 19 subjects for the PNES, and 20 for HC. Additionally, we randomly divided each subject into two parts, respectively, 70% and 30%. In k-fold cross-validation, one subject of each condition is tested in every fold, and all subjects are tested exactly once after the k-fold cross-validation. During the k-fold training, the algorithm, for each fold, assigns a label to each chunk of data using statistical rules. This process is repeated for each of the 8 k-fold tests, and the accuracy is the is computed as the average of each fold accuracy score. In this way, the training and test set for each fold do not contain instances from non-overlapping subjects, which is the best solution to avoid building a classifier with better generalization in identifying PNES features from HC. It is worth mentioning that MLP parameters were inferred using a trial-and-error approach, commonly used for optimization. Furthermore, we use the *F1 score* measure, which includes precision and recall information (see Equation (12)), to infer our consideration on classifiers performance. In Table 2 we report the outcomes of our binary classification. Our MLP classifier got an *F1 score* of 86.75% in PNES classification and 71.22% in HC classification, respectively. Recall and precision are, respectively, 96.73% and 56.42% for PNES and HC, whereas precision is 78.23% for PNES and 91.23% for HC. Doing a comparison between SVM and LDA, in terms of *F1 score*, they report 87.44% and 83.26%. The LDA classifier achieved the worst F1 when compared with MLP and SVM.

**Figure 9:**
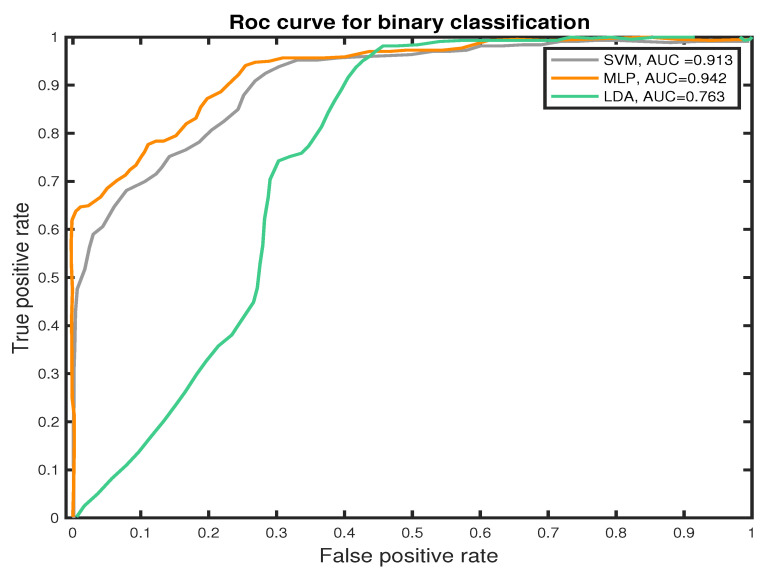
ROC curves of the proposed SVM, LDA, and MLP classifiers for binary classification task (PNES versus HC).

We measure the performance of the classifiers using *TP*, *TN*, *FN*, and *FP* and the receiver operating characteristic (ROC) curves to present the results. The ROC curve is used to plot the probability of correct classification (sensitivity) against the probability of wrong classification (1-specificity) at various threshold settings. Thus, each point within the ROC curve represents a sensitivity/specificity pair for each threshold. The performance of the classifier is measured by the area under the ROC curve. MLP classifier shows the best performance score in PNES versus HC patient-based classification, which estimates the probability that subject *j*th (*j* = 1,..., 39) belongs to class PNES or HC, based on how the epochs were labeled by the classifier. Experimental results show that the MLP outperform the other approaches in classifications of PNES versus HC, achieving an averaged accuracy of 89.7%. This result was confirmed also by the area under the curve (AUC) for the Receiver Operating Curve (ROC). As reported in Figure 9, the MLP classifier shows the highest AUC value. The area under the curve measures the ability of the classifier to correctly identify those subjects with PNES from those with HC. The best classification performance, as measured by the AUC (see Figure 9), is obtained with MLP (AUC = 0.942). Additionally, we highlight an AUC of (AUC = 0.913) for SVM and (AUC = 0.913) for LDA.

## 6. Discussion

The present research addressed the challenging issue of identification robust PNES electrophysiologic-markers in rest-EEGs. We approached EEG signals by using PSD and PLI-based graph measures. Additionally, we addressed the challenge to select statistically significant features for an automatic machine learning-based classification.

Different cortical brain regions are toned to oscillate at an individual rate [56]. These oscillations reflect local, intrinsic physiological mechanisms related to the fine-tuning of corticothalamic circuits within each cortical region tended to resonate at approximately the same frequency. Rhythmic patterns of neural activities are believed to play an important functional role in local processing and communication between different neuronal systems. Thus, the study of brain frequencies in PNES does not only have theoretical relevance but also clinical implications. In addition, we used PSD to find specific differences in the modulation of tuning frequency of brain areas among PNES subjects and healthy controls [57]. We further investigated the functional connectivity in brain networks of PNES patients and healthy controls. The functional network represents the temporal lag of phase in the brain network communication, and the network measures are tools used to investigate the efficiency of communication between brain regions. The human brain can be described as a small-world network, that is structured around a large number of spatially distributed network communities with clustered connectivity, in which the local computations are likely to be highly segregated. Integration of segregated information in small-world is promoted by network hubs, which connect network communities and ensure efficient communication and information integration. Our results are discussed in the next subsections.

We used three machine-learning algorithms, namely Support Vector Machine, Linear Discriminant Analysis, and Multilayer perceptron, to disentangle PNES versus HC from graph-based indices.

### 6.1. PSD Measures

In Figure 4, we check for a spatial pattern of PSD to infer brain areas with strong differences across PNES and control subjects. Particularly, we found that, in central and parieto-occipital areas, the PSD of healthy subjects was higher than those of PNES subjects. We hypothesize that these results in PNES may be related to weakness in information processing and network connections and that the neural model of PNES subject misses to engage processing in long and in short-range. On the other hand, an increase of PSD in delta band in frontal and central areas for PNES may reflect a lack of influence of sub-cortical structures on cortical activity [58]. Frontal and central areas are implicated in the control of sensory feedback and attention. The hypothesis that, in subjects with PNES, there is a greater increase in power in these areas leads us to think that there could be a greater alert and pre-activation of the cortical circuit implicated with sensory and movement control. Thus, the pre-activation or hyper-activation in this cortical circuit could leave this to fire and trigger a long front-to-back route when activated. Our findings, supported by other studies [26,27], highlighted that, in PNES EEGs, there are network alterations more related to high-frequency oscillations. Additionally, we found an overall alpha rhythm weakness in PNES when compared to HC, whereas a strengthening PNES is more than HC in delta and theta band. Therefore, alpha alterations could reflect pathological resting-state dynamics in which thalamus, midline, frontal, and parietal cortices play an important role in accountability to seizures [59]. The alpha rhythm is thought to arise through cortico-thalamic interactions, and to possibly reflect top-down processes that subtend a vast number of cognitive operations, particularly attention, working memory, and sensory system control [60]. Arıkan et al. [27] found that PNES correlates with high-frequency oscillations on central and parietal areas, whereas Meppelink et al. [26] found a decrease in PSD in the beta band.

### 6.2. PLI Measures

In Table 1, we present our post-hoc analysis to highlight differences in delta, theta, alpha, and beta band. These results may suggest frequency-specific network organization and imply various functional roles for different frequency bands. Recently, scalp EEG-based studies revealed that PNES might lack a relatively long linkage in the brain network topology, indicating the impairment of information transferring and processing [11]. As shown in Figure 5, Figure 6 and Figure 7, the PNES versus HC network topologies differ in the bilateral hemisphere with a marked difference in alpha and beta band. Decreased functional connectivity in the alpha band could be associated with a decreased attitude to generate seizure, thus uncontrolled movements triggered by altered neurobiological substrates. This hypothesis could be strengthened by the finding that PNES patients exhibited a more path-like topology with a decreased long-range synchronization in the alpha and beta band. Moreover, the network properties are effective to capture the information of spatial differentiation in the networks. The network synchronization maps in Figure 5, Figure 6 and Figure 7 shows that subjects with PNES have fewer and weak links between the frontal and the temporal/occipital areas, which is consistent with the structural abnormality difference between the two groups found in References [61,62]. The working mechanisms of PNES are further revealed in Figure 5, Figure 6 and Figure 7, which shows that the PLI analysis captures spatial patterns of anomalous network information within brain networks. These results corroborate the hypothesis that PNES is a phenomenon of neuronal disconnection and that, in high frequency, integration and differentiation of the network are lost.

### 6.3. Measures of Segregation and Integration

In this paper, we used *CC*, *Ge*, and *SPL* to evaluate segregation and integration. The box plots in Figure 8 shows how the PNES subjects have increased clustering coefficients and decreased shortest path length when compared to the normal subjects; this could be a predictor of impaired local information processing and loss of global integration in patients [63]. In PNES, the higher clustering values are a feature of regular local networks with symmetrical structures and higher connections with their closest neighbors. Instead, short path length describes the number of edges among vertices, thus being used as a feature for random networks. PNES has been shown to correlate with functional and structural brain network alterations using MRI-related studies [19]. We also found a decreased global efficiency that could reveal that PNES might lack a relatively long linkage in the brain network topology, indicating impairment of information transferring and processing. In practice, PNES patients can be easily discerned from normal subjects based on their abnormal behavior. The *CC*, *Ge*, and *SPL* are measurements determined by spatial topology where the complete information of a network is significantly more complex than the adopted statistical measurements. Thus, the essential spatial information of a network would be meaningful to further improve the ability to differentiate the PNES subjects. In detail, we found a decreased averaged shortest path length (*SPL*) in PNES for all the EEG sub-bands under consideration. Further we found a decreased *Ge* in the *delta*, *theta*, and *alpha* bands, indicating the network of PNES subjects, such as a model highly segregated and unable to integrate information over long distances. The general hypothesis is that to process relevant information the neural responses are not only reflected by a change of neural activity, in certain regions of the brain, but also by a global reorganization of connectivity patterns. We highlight a loss of network synchronization (see Figure 8), such as a spatial pattern related to a change of neural activity in certain brain regions involved in a global reorganization of connectivity patterns during the information processing. We can evaluate the ability of the brain to encode information using *CC*, *Ge*, and *SPL* measures, as well to characterize the effective integration of distributed information across the whole brain (Figure 8). The measures of connectivity, calculated as the strength of the edges between the nodes, highlight loss of functional integration between brain regions due to the loss of long-range linking. In addition, our results are in agreement with other studies [15,16,18]. Differently from Xue et al. [11], we report cluster coefficient values higher in PNES than HC. However, we also observe that PNES subjects had higher *CC* more than HC.

### 6.4. Measures of Centrality

We used Node Betweenness, such as a centrality measure, to characterize the relevance of individual nodes in the network. We found that HC subjects have a higher level of *NB* than PNES subjects, except for the beta band. Furthermore, our analyses suggest that impaired information processing involves connectivity across multiple cortical areas in the beta bands. Moreover, in the beta band, we found fewer brain connections in the right frontocentral cortical areas for PNES. Our findings are coherent with previous literature evidence, pointing to the pathogenesis of PNES disorders [28,64,65].

### 6.5. Small-Worldness

In order to further understand if clustering coefficient or path length are contributing equally to the observed small-worldness differences between the group, we performed a small-world analysis. The small-world networks have a high clustering and a short path length, as in random networks. Given that the small-world model supports both specialized and integrated information processing in the brain, we attempt to describe the effect of the impairment on the topological property of small-worldness characteristics derived from EEG connectivity data. Furthermore, the PNES group presents a significant network differentiation in theta, alpha, and beta bands, with fewer small-worldness characteristics. As reported in Ding et al. (2013), the small-worldness in PNES has altered functional and structural connectivity networks, which is related to a more regular organization in large-scale brain networks [19]. Additionally, Ding et al. founded alteration in region involving attention, sensorimotor, sub-cortical networks [19]. In other studies, the loss of integration and segregation in PNES was supposed to be related to emotion, executive control, and motor function impairments [18].

### 6.6. Classification

The prediction of PNES from rest-EEG signals is a challenging task. In this paper, we compute several network features from resting-state EEG activity and evaluate if these can be used for the identification of PNES. In this paper, we computed a power spectrum distribution analysis in the range 0.5–32 Hz, further split into four sub-bands, namely delta, theta, alpha, and beta. Additionally, we analyzed our rest-EEG data using a functional connectivity approach. From the phase lag index mathematical framework were computed several graph indices in four frequency bands. Our results suggest PSD analysis be less efficient than PLI in providing insights to distinguish PNES from HC. Additionally, our analysis suggests that network measures are more statistically robust in PNES versus HC discrimination than those measures extracted from PSD, which, instead, turns out to be less discriminative. Thus, to increase the classification performance in our automatic group discrimination framework, we used the more statistically robust feature, thus the graph-based indices. However, it is noteworthy to mention that the PLI-based graph indices are more computationally demanding than PSD; thus, from a practical point of view, they could be time-consuming. Graph indices turn out to be more specific and physiologically meaningful, less prone to noise and volume conduction confounding factors. Additionally, we attempt to infer insight in rest-EEG data; thus, the background activity could somehow be influenced by the subject brain state during the recording and other additional confounding factors. We note that graph-based index could be used to infer physiological mechanisms behind PNES, thus being a good feature to discriminate between two groups. In literature, we found previous studies that suggest how somehow PLI systematically failed to produce accurate classifications between healthy and epileptic groups. In Reference [66], authors use extreme learning machine (ELM) algorithms to identify healthy and epileptic subjects from brain activity measured with MEG in a resting state. In this paper, they train ELM on PSD, PLI, and PLV handcrafted features. They found that features based on PSD achieve higher results in classification more than PLI and PLV.

Differently, we prove how to graph-based indices, extracted from PLI network could represent a robust feature for PNES versus HC discrimination in rest-EEGs. Here, we used three different machine learning algorithms; SVM, LDA, and MLP that are able to discriminate PNES versus HC in a graph-based indices dataset. In this paper, we used an 8 k-fold cross-validation approach to train the proposed ML classifiers. The average performance is measured with commonly used measures as reported in Section 5.3. Our results highlight an Area Under the Curve of 0.763 for LDA, 0.913 for SVM, and 0.942 for MLP. In terms of AUC, the MLP classifier outperforms both SVM and LDA classifiers. Additionally, SVM achieves a Precision of 77.74% in PNES discrimination and 89.22% in HC, which is lower than MLP. Our results show that MLP achieve higher Precision, Recall, and *F1 score* when compared to SVM and LDA classifiers.

## 7. Conclusions

In this study, we succeeded to detect the PNES disorder form the rest-EEG signal. The methodology we proposed uses Power Spectral Density to reveal two main characteristics that differentiate PNES cases from Healthy Controls (HC). First, PNES patients have an increased spectral power density in the delta and theta bands in the frontal and central areas. Second, they present an overall decreased spectral power in the other frequency bands under analysis. Additionally, we noted that functional connectivity networks exhibited altered nodal characteristics in global efficiency, node betweenness, and path length. We also found a decreased coupling strength of functional connectivity in PNES. The synchronized oscillatory maps showed high sensitivity to differentiate PNES patients from healthy controls. This supports the hypothesis that the PNES reflects an irregular brain network with a loss of functional connectivity and a disturbance of the right back-to-front, or vice-versa, patterns of information flow in brain areas related to cognitive operations, attention, working memory, and movement regulation. Additionally, as reported in Table 1, we found that the shortest path length and node betweenness are the most significant/discriminative indices with differences within high frequency in the central and posterior areas. Differences in network parameters could highlight impairment in segregation and integration, as well as in information processing, during rest-activity. These alterations could also be related to the psychological condition or stress induced by the transient EEG monitoring event, as well as the alertness of the subject waiting for a particular/apparent trigger. From this perspective, functional connectivity analysis, more than PSD, might be effectively used to discriminate PNES in scalp EEG time series. In future work, we will attempt to increase the cohort of PNES subjects, as well as to collect EEG data with a high-density EEG system, to raise the statistical precision of our analysis. It could also be interesting to investigate whether the abnormal organization found in this study can be further confirmed in a large cohort of PNES subjects using other methods, such as weighted PLI or directed PLI. In this paper, we also attempt to find a combination of features that allow to discriminate PNES from HC. However, it is less evident how to identify features, to be used in machine learning algorithms. To this end, we statistically evaluated two set of features extracted, respectively, using PSD and PLI. The latter showed a more statistical relevance in discriminating PNES from HC, based on signatures of brain network alteration. Thus, we attempt to use those features for an automatic ML-based classification with the main goal to highlight how commonly used classifier are suitable to infer classification from network indices. Here, we proposed Support Vector Machine, Linear Discriminant Analysis and Multilayer perceptron. We used a k-fold validation for subject discrimination. In our analyses, MLP outperforms LDA and SVM in PNES discrimination, achieving an accuracy of 91.02% when compared with SVM (79.41%) and LDA (76.72%). In conclusion, our framework, based on a graph indices features and common ML classifiers, turn out to be a robust way to approach the challenge in PNES discrimination. In addition, in order to exploit brain state changing in PNES, we plan to apply this method to data acquired during transcranial magnetic stimulation (TMS) [67], as well to compute source modeling, to study and map how the brain networks of PNES change under external perturbations.

## Figures and Tables

**Table 1:** The table report results from a statistical analysis across cluster of EEG sensors (see EEG layout parcelization in Section 5.1) in all of the frequencies band under analysis. We evaluated Global efficiency, Node betweenness, Cluster coefficient, Small world, and Shortest path characteristic. We performed a post-hoc analysis for multi-comparison. The *p*-value (*p* < 0.05) corresponds to significant difference in network index values for the testing conditions. See Section 3.7 for further information.

	Frontal Area				Central Area				Parieto-Occipital Area			
Graph Index	*δ*	*θ*	*α*	*β*	*δ*	*θ*	*α*	*β*	*δ*	*θ*	*α*	*β*
Global efficiency	0.0532	0.132	0.102	0.251	0.04	0.06	0.0128	0.0013	0.073	0.122	0.016	0.00123
Node betweenness	0.273	0.322	0.421	0.151	0.073	0.122	0.033	0.023	0.013	0.018	0.263	0.032
Cluster coefficient	0.074	0.126	0.452	0.321	0.142	0.785	0.0412	0.022	0.561	0.174	0.0430	0.0012
Small world	0.752	0.134	0.144	0.434	0.33	0.431	0.04	0.014	0.335	0.453	0.034	0.174
Shortest path	0.331	0.123	0.424	0.041	0.021	0.014	0.143	0.041	0.012	0.033	0.012	0.044

**Table 2:** Binary classification performance (*Precision*, *Recall*, *F1 score*, and *Accuracy*) of SVM, MLP, and LDA.

*Precision*	*SVM*	*MLP*	*LDA*
PNES	77.74%	78.23%	75.42%
HC	89.22%	91.23%	93.42%
***AVG***	83.48%	**85.73**%	84.42%
***Recall***			
PNES	95.24%	96.73%	96.42%
HC	54.22%	76.42%	63.12%
***AVG***	74.73%	**86.57**%	79.77
***F1 score***			
PNES	87.44%	86.75%	83.26%
HC	65.61%	71.22%	65.07%
***AVG***	76.52%	**78.98**%	74.16%
***Accuracy***			
PNES	89.21%	96.82%	82.16%
HC	69.61%	85.22%	71.27%
***AVG***	79.41%	**91.02**%	76.72%
